# Dr. R. C. Word

**Published:** 1890-08

**Authors:** 


### DR. R. C. WORD.

It is with sorrow that we are again called upon to chronicle the death of one of Atlanta's old and honored physicians. Dr. R. C. Word died in Decatur, Ga., on the 20th day of July, after an illness of several months. For many years he successfully edited the *Southern Medical Record*, of this city, and resigned the Editorial chair but a few months before his death. He was Professor of Physiology in the Southern Medical College, a position which he filled with eminent success. As a practitioner, he enjoyed the love and confidence of a large circle of patients. As a Christian gentleman, he won the respect and admiration of all who knew him. He leaves behind him the record of a life's work well done.

---

Dr. W. F. Westmoreland has been elected to the Chair of Surgery in the Atlanta Medical College, left vacant by the death of his father.

